# GLYNAC SUPPLEMENTATION IN OLDER ADULTS IMPROVES GLUTATHIONE, MITOCHONDRIA, AGING HALLMARKS, AND FUNCTION

**DOI:** 10.1093/geroni/igae098.0656

**Published:** 2024-12-31

**Authors:** Rajagopal Sekhar, Premranjan Kumar, George Taffet, Charles Minard, James Suliburk, Farook Jahoor, Chun Liu, Raja Muthupillai

**Affiliations:** Baylor College of Medicine, Houston, Texas, United States; Baylor College of Medicine, Houston, Texas, United States; Houston Methodist Hospital, Houston, Texas, United States; Baylor College of Medicine, Houston, Texas, United States; Baylor College of Medicine, Houston, Texas, United States; Baylor College of Medicine, Houston, Texas, United States; Baylor College of Medicine, Houston, Texas, United States; Baylor St. Luke’s Hospital, Houston, Texas, United States

## Abstract

Elevated oxidative stress (OxS), mitochondrial dysfunction, and aging hallmarks contribute to aging, but improving/reversing these defects in older adults (OA) has been challenging. We identified that deficiency of the intracellular antioxidant glutathione (GSH) could play a role and reported that supplementing GlyNAC (combination of glycine and N-acetylcysteine [NAC]) in aged mice improved GSH deficiency, OxS, mitochondrial fatty-acid oxidation (MFO), and insulin resistance (IR). To test whether GlyNAC supplementation in OA could improve GSH deficiency, OxS, mitochondrial dysfunction, IR, aging hallmarks and physical function, we conducted a placebo-controlled randomized clinical trial in 24 OA and 12 young adults (YA). The OA was randomized to receive either GlyNAC or placebo for 16-weeks, and the YA received GlyNAC for 2-weeks. Participants were studied before, after 2-weeks, and after 16-weeks of supplementation to assess changes in intracellular GSH concentrations, OxS, MFO, molecular regulators of energy metabolism, inflammation, endothelial function, IR, multiple aging hallmarks, gait speed, muscle strength, 6-minute walk test, body composition, and blood pressure. We found that compared to YA, the OA had GSH deficiency, elevated OxS, mitochondrial dysfunction (with multiple defects in molecular regulation), inflammation, endothelial dysfunction, IR, multiple aging hallmarks, impaired physical function, and increased waist circumference and systolic blood pressure. GlyNAC (and not placebo) supplementation in OA improved/corrected these defects. In conclusion, GlyNAC supplementation in OA for 16-weeks was found to be safe, well-tolerated, and effective in its ability to reverse multiple age-associated declines and age-related abnormalities. GlyNAC supplementation in older humans is an effective nutraceutical to promote healthy aging.

